# Utilization of animal models to investigate nonalcoholic steatohepatitis-associated hepatocellular carcinoma

**DOI:** 10.18632/oncotarget.8641

**Published:** 2016-04-07

**Authors:** Jian Wu

**Affiliations:** ^1^ Key Laboratory of Molecular Virology, Fudan University Shanghai Medical College, Shanghai, China; ^2^ Shanghai Institute of Liver Diseases, Fudan University, Shanghai, China

**Keywords:** nonalcoholic fatty liver disease, end-stage liver disease, nonalcoholic steatohepatitis (NASH), NASH-associated hepatocellular carcinoma (NASH-HCC)

## Abstract

Nonalcoholic fatty liver disease (NAFLD) comprises a spectrum of liver disorders with fat accumulation from simple fatty liver, nonalcoholic steatohepatitis (NASH), fibrosis/cirrhosis and NAFLD/NASH-associated hepatocellular carcinoma (HCC). NASH is a progressive form of NAFLD and requires medical attention. One of 5-10 NASH patients may progress to end-state liver disease (ESLD or cirrhosis) in 5-10 years; meanwhile, life-threatening complications of ESLD and HCC account for major mortality. An increasing burden of NAFLD in clinics, elucidation of its pathogenesis and progression, and assessment of the efficacy of potential therapeutics demand reliable animal models. Most NASH-associated HCC occurs in cirrhotic subjects; however, HCC does appear in NASH patients without cirrhosis. Lipotoxicity, oxidant stress, insulin resistance, endoplasmic reticulum stress, altered adipokine and lymphokine profiles and gut microbiome changes affect NAFLD progression and constitute key pathobiologic interplays. How these factors promote malignant transformation in a microenvironment of steatotic inflammation and fibrosis/cirrhosis, and lead to development of neoplasms is one of critical questions faced in the hepatology field. The present review summarizes the characteristics of emerging rodent NASH-HCC models, and discusses the challenges in utilizing these models to unveil the mysteries of NASH-associated HCC development.

## INTRODUCTION

Nonalcoholic fatty liver disease (NAFLD) covers a wide spectrum of disorders with fat accumulation, including simple fatty liver (SFL), a progressive form of nonalcoholic steatohepatitis (NASH), fibrosis/cirrhosis and NASH-associated hepatocellular carcinoma (NASH-HCC). NAFLD has become prevalent in many developed and developing countries [1], and approximately 1.0 billion individuals are affected worldwide, ranging from 10 to 36% of the general population [1, 2]. Up to 70-80% of type II diabetic patients have various stages of NAFLD [3], and there appears to be a genetic basis for the severity of NAFLD in diabetic patients [4]. NAFLD also affects young children and adolescents, and it has become the most common liver disorder in the pediatric population [5]. It is estimated that 13% of children and adolescents in the US are affected with NAFLD, and that 23% of the subjects with NAFLD have evidence of steatohepatitis and bridging fibrosis or cirrhosis was observed in 9% of the children with NASH [5].

**Figure 1:**
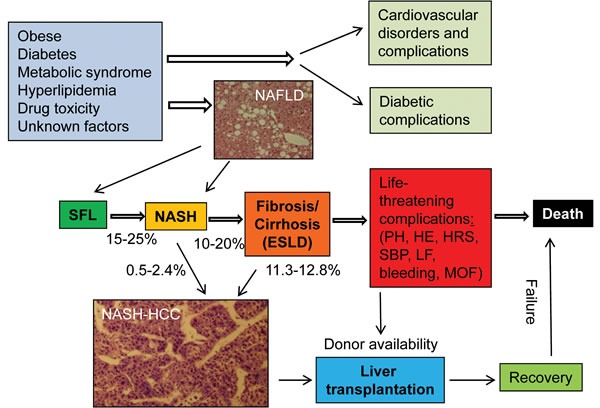
Development, progression and prognosis of NAFLD from SFL to end-stage liver disease (ESLD) in humans Cardiovascular and diabetic disorders and their complications may account for a large portion of morbidity and mortality in patients with obese, diabetes and metabolic syndrome. A micrograph of NAFLD was obtained from an individual who had an abdominal surgical procedure with permission. A histologic micrograph of NASH-HCC was from a pathologically confirmed NASH patient after surgical resection. The collection of patient specimens was approved by the institutional ethic committee. Liver transplantation is the only established therapy for ESLD and HCC. Given the scarcity of donor livers, only a small fraction of candidates qualified for liver transplantation will eventually receive transplantation before deterioration. Life-threatening complications of ESLD include portal hypertension (PH), liver failure (LF), bleeding, hepatic renal syndrome (HRS), hepatic encephalopathy (HE), spontaneous bacterial peritonitis (SBP), and multiple organ failure (MOF). The numbers besides arrows in the figure are the rates of possible transition or transformation. NAFLD = nonalcoholic fatty liver disease; NASH-HCC = nonalcoholic steatohepatitis-associated hepatocellular carcinoma; NASH = nonalcoholic steatohepatitis; SFL = simple fatty liver.

In general, SFL is reversible with proper diet control and exercise, whereas NASH requires medical attention, and may progress to end-stage liver disease (ESLD) from which HCC may develop. It has contributed to an increased HCC incidence in the US. Both NASH-associated ESLD and HCC constitute NAFLD-associated mortality although cardiovascular and metabolic abnormalities are the leading complications of NAFLD-related mortality [6]. Clinical investigation suggests that 15-25% of SFL may progress to NASH, and 10-20% of NASH patients may advance to ESLD in 5-10 years [7, 8], which represents a large portion of crytogenic cirrhosis. Moreover, there often are no obvious signs of hepatic dysfunction prior to occurrence of liver failure, HCC or other life-threatening complications [6, 9]. NASH-associated ESLD has already become the major indication for liver transplantation in the US [10]. Once cirrhosis develops, the rate of HCC in cirrhotic patients was reported to be about 11.3 % in Japanese in 5 years [7], or 12.8% in 5 years in the US [11]. Special attention must be paid to NASH patients without advanced fibrosis or cirrhosis because HCC does occur at this stage, and once it occurs, the malignancy often presents with a larger tumor size than in cirrhotic patients [12], although the rate is thought to be relatively low (0.5-2.4% over 7 years in different ethnic groups and regions) [2, 7, 11] (Figure 1). From limited clinical data, it appears that histopathologic features, risk factors and prognosis of HCC occurring in NASH-associated cirrhosis are significantly different from the features of other etiologies of liver disease, such as hepatitis B or C viral infection [13]. Moreover, the pathogenic factors contributing to the oncogenic process in NASH-HCC appear to differ from HBV or HCV infection, and are as yet largely undefined [7].

The clinical studies on NASH-associated HCC (NASH-HCC) are in an initial stage, focusing on epidemiologic and demographic investigations, such as the incident of HCC occurring in NASH-associated cirrhosis or in any stage of NASH without cirrhosis, and on clinical manifestations and histopathologic features of NASH-HCC in comparison with HCC derived from etiologies of other liver diseases [2, 6, 8, 13]. One focus that clearly demonstrates the impact of NASH-HCC is the candidate profile shift for liver transplantation. HCC secondary to NASH has becomes the second most common etiology of HCC leading to liver transplantation in the US [10]. There are many unsolved issues facing the increased incident of NASH-HCC in clinical practice, such as practice guidelines for screening, as well as diagnosis and management of HCC occurring with or without cirrhosis in NASH patients [14]. For such clinical challenges, long-term follow-up of this category of patients and well-planned multicentric observations will hopefully establish feasible solutions. Although deep sequencing of patient samples have identified genetically predisposing factors, e.g., the PNPLA3 rs738409 C > G polymorphism increases the risk of progressive steatohepatitis, fibrosis and NASH-related HCC, it is very difficult to establish a causative effect in such a study [15]. Other studies involve metabolomic analysis of blood or biopsy specimens from obese, SFL, NASH, fibrosis/cirrhosis and HCC, and attempt to discover novel molecules critically governing malignant transformation and progression [16]. Another area of active research employing a systems biology approach has discovered the clues of altered gut microbiota for NASH development and possible link to gut and liver malignancies through bile acid metabolites, such as increased levels of deoxycholic acid, which is toxic to hepatocytes and provokes senescence of hepatic stellate cells (HSC). Tumorigenic factors, such as platelet-derived growth factor (PDGF) and vascular endothelial growth factor (VEGF) released from senescent HSCs facilitate hepatocellular transformation to malignancy in mice [17–19]. These clinical or translational studies provide valuable hints of possible factors or pathways involved in NASH development and progression at population or individual levels. Nevertheless, we are facing a variety of challenges when approaching NASH-HCC in both clinic and laboratory investigations (Key Point 1), and it is conceivable that translational studies without valuable animal models will leave many critical questions unanswered due to ethical concerns.

Key Point 1 Challenges in investigating NASH-HCC

- NASH-HCC is an emerging challenge in the hepatology field
- Single etiology vs. multiple factors contributing to the pathogenesis of base diseases
- Population studies could identify risk factors but hardly establish solid causative-effect ties
- Lack of reliable NASH animal models is a major problem

## CHALLENGES IN INVESTIGATING NASH-HCC

### NASH-HCC is an emerging challenge in the hepatology field

Unlike other base liver disorders, such as viral hepatitis, alcoholic liver disease or genetic deficiencies, NASH-associated HCC (NASH-HCC) was not recognized until only a few years ago [20]. Until now, few studies focusing on the development of NAFLD/NASH with HCC have been conducted [7, 21]. In contrast, over decades, the molecular pathogenesis of HBV or HCV infection and its contribution to HCC development have been extensively studied employing both clinical and laboratory approaches [22]. Although the exact molecular pathogenesis of HCC development in these viral liver diseases have not been fully revealed, tremendous progresses have been made in terms of clinical features, managements, oncogenic potential of viral proteins, and the critical role of viral genomic integration into the host genomes, as well as positive correlations between success of anti-viral therapy and decrease in HCC incidence [23–27]. In contrast, basic and clinical studies of NASH-HCC are in their infancy [17], and many clinical issues and basic understanding of NASH-HCC pathogenesis remain unexplored and elusive.

### Single etiology *vs*. multiple factors contributing to the pathogenesis of base diseases

HBV or HCV infection, alcoholic liver disorders, and inherited deficiencies, such as α1-antitrypsin deficiency, Wilson disease or hemochromatosis, are caused by single etiological factors or genetic mutations. In contrast, NAFLD is a complication of various metabolic disorders, including obesity, diabetes, metabolic syndrome and hyperlipidemia, etc. [8]. Fatty accumulation in the liver is the hallmark of a series of pathophysiologic conditions, and could also be the toxic consequence of various medications [28]. In fact, the pathophysiology of NAFLD/NASH differs from patients to patients, and multi-factorial features can present in a single NAFLD/NASH patient, which adds extra layers of complexity to etiologic analysis when dissecting risk factors or possible pathologic mechanisms of HCC in NAFLD/NASH [7, 8].

### Population studies could identify risk factors but hardly establish solid cause and effect ties

Longitudinal studies indicate that HCC prevalence is approximately 0.5% in steatosis and up to 2.4% in NASH (Figure 1). In this context, it is thought that HCC tends to be an infrequent complication of NASH. However, the high prevalence of NAFLD confers that NAFLD-related HCC contributes significantly to the disease burden [20]. To establish the link between NAFLD/NASH with HCC in population studies, four clinical approaches are often undertaken, including: 1) Well-documented case reports; 2) Retrospective studies demonstrating that HCC is developed from cryptogenic cirrhosis, implicating that NAFLD is a precursor of HCC; 3) Prospective studies that evaluate late complications from NAFLD patients; and (4) Prospective studies that intend to follow up a large cohort of subjects with NASH and to determine metabolic abnormalities, progression of NAFLD/NASH and occurrence of HCC for a long period. Undoubtedly, these clinical studies are of paramount importance in gathering epidemiologic data, identifying biomarkers that could distinguish when HCC occurs in NASH or the cirrhotic liver, and assessing the effectiveness of therapeutic algorisms or exploring new therapeutic interventions [8]. Nevertheless, these clinical studies are less likely to provide lines of valuable evidence that demonstrates how risk factors or macro- and microenvironmental alterations facilitate the transformation of steatotic hepatocytes with dysplasia [29] to malignant cells, or normal liver progenitor cells to cancer stem cells (CSCs) or tumor-initiating cells (T-ICs). CSCs or T-ICs are thought to be responsible for oncogenicity, progression, relapse, resistance to therapeutics and metastasis [30]. Such approaches can only be undertaken in experimental animal models with acceptable ethical standards.

### Lack of reliable NASH animal models is a major problem

There exist a number of rodent models of NASH available [31–33], and characteristics of each model are extensively reviewed, including genetic background, natural occurring or diet induction (diet types, feeding duration), obese status, insulin resistance, adipokine profile, inflammatory activity, steatotic, necroinflammatory and fibrotic extent, malignant intensity, etc. [34–36]. These overviews provide a first line of information when one considers using these models. However, none of these models truly reflects the disease course, pathologic feature of molecular interplays, and histologic characteristics of NASH in patients due to the diversity of the disease spectrum [34–37]. These rodent models are often induced in 2-4 months with various degrees of steatosis with or without significant necroinflammatory changes. Fibrosis is often mild if there is any, and HCC rarely develops in such a short duration of induction by high fat/Calorie (HFC) with fructose or methionine/choline-deficient (MCD) diet. HCC commonly develops in mice fed HFC or fast-food-type diet in approximate 12 months. Moreover, neoplastic nodule numbers, size (macroscopic or microscopic) and malignant degree (from dysplasia, adenoma to carcinoma) vary from mouse to mouse and are often unpredictable [38]. These variables make such an experiment very costly. For these reasons, investigators are seeking more acceptable models by reducing the duration of induction from steatosis to HCC occurrence.

## CHARACTERISTICS OF CURRENTLY AVAILABLE RODENT NASH-HCC MODELS

Depending on the approaches used for the induction of NASH and subsequent HCC, currently available models of primary HCC from NASH are classified into genetic manipulation, diet induction and carcinogenic exposure. Genetic manipulation includes phosphatase and tensin homolog (PTEN) knock-out NASH-HCC mouse model [39], argumenter of liver regeneration (ALR)-knock-out mouse model, knock-out of melanocortin 4 receptor (MC4R) [40] and the 129S1/SvImJ strain of mice with a metabolic iron abnormality [41]. Diet induction varies from the STAM model to MCD, HFC diet, choline-deficient high fat diet (CD-HFD), choline-deficient amino acid-defined (CDAD) or an American (sedentary) lifestyle-induced obesity syndrome (ALIOS) model. A NASH model plus carcinogenic exposure is another category of NASH-HCC, such as MCD or HFC diet plus diethylnitrosamine (DEN) [42]. By employing a series of lines of knock-out mice, including Rag1−/−, CCR2−/−, Ltbr^Dhep^, and IkkB^Dhep^ to determine the critical role of intrahepatic CD8+ T cells and NKT cells in the development of NASH-HCC in mice fed CD-HFD, the investigators have employed an innovative approach to dissect LIGHT, the ligand of LTβ receptor (LTβR) (derived from NKT cells) and lymphotoxins released from these two subsets of lymphocytes in the transition from NASH to HCC [43]. Endoplasmic reticulum (ER) stress plays a crucial role in the mediation of lipotoxicity and insulin resistance, and may cause ballooning degeneration of hepatocytes. The induction of *in vivo* ES stress by deleting MUP in MUP-uPA knock-out mice conferred a venue to explore its contribution to NASH development and malignant transformation [44]. The use of an implanted xenograft in fatty liver may determine whether fatty liver or steatohepatitis promotes tumor growth or metastasis. It is our intent to summarize the main characteristics of each NASH-HCC model (Key point 2), and provide guidelines in selection of available models for particular research questions.

Key Point 2 Classification of rodent NASH-HCC models

- Genetic manipulation by site-specific knock-out of a gene or in combination of genes.
- Induction of NASH-HCC by various formulas of diet, such as HFC, MCD, CD-HFD, CDAD, STAM or ALIOS
- NASH model plus a carcinogen exposure, i.e. diethylnitrosamine
- Implantation of xenografts in a fatty liver for examining tumor growth and metastasis

### Employing genetic manipulation to understand the function of critical genes in NASH-HCC progression

#### PTEN knock-out mice

PTEN is a ubiquitously expressed tumor suppressor gene. Its expression is reduced or absent in HCC. Reduced PTEN expression correlates with increased tumor grade, advanced stage and poor prognosis. A liver-specific knock-out using a “cre-lox” technology with the albumin promoter led to severe steatohepatitis and HCC between 10 and 40 weeks in PTEN null mice [45]. With this model, it has been demonstrated that eicosapentaenoic acid (EPA), a poly-unsaturated fatty acid (PUFA), protected these mice from developing steatohepatitis and subsequent HCC during a 40-week intervention. The antioxidant stress, inhibition of MAPK activity and a low ratio of oleic to stealic acid are believed to be the underlying mechanisms of protection [46]. A subsequent study showed that combination of knock-down of both PTEN and glucose-regulated protein 78 (GRP78), a chaperone protein, could exacerbate steatohepatitis and accelerate the malignant transformation in the liver of PTEN/GRP78 null mice. These double knock-out mice develop both HCC and cholangio-carcinoma, indicating that GRP78 is protective and could be a novel regulator for PTEN-deficiency-mediated liver injury and cancer progression [47].

#### Lack of ALR promotes the development of NASH and HCC

ALR is a pleiotropic protein originally defined as a growth factor critical for regeneration after partial hepatectomy, and its levels are lower in livers with steatotic alteration, such as alcoholic or nonalcoholic steatohepatitis, than in controls. However, its biochemical function was unclear until a line of liver-specific ALR knock-out (ALR-KO) mice was generated [48]. These ALR-KO mice exhibit excessive hepatic steatosis with significant inflammation, abnormal mitochondrial respiratory function and increased oxidative stress. These mice also have lower liver levels of carbamyl-palmitoyl transferase 1α and ATP synthase subunit ATP5G1, which may be responsible for the defects in mitochondrial fatty acid transport and ATP synthesis. At 4-8 weeks of life, liver inflammation became more severe with hepatocellular necrosis, ductular proliferation and fibrosis. HCC developed in nearly 60% of the mice by 1 year after birth [48]. It is speculated that HCC develops in severe steatotic and inflamed liver with increased oxidant stress as a result of loss of ALR function in preventing ROS generation in the mitochondrial respiratory chain. Hence, this model could advance our understanding regarding the critical role of mitochondrial energy metabolism and oxidant stress in the transition from hepatic necroinflammation, steatohepatitis, and fibrogenesis to carcinogenesis.

#### MC4R-deficient mice

MC4R is a seven-transmembrane G protein-coupled receptor that is expressed in the hypothalamic nuclei and is thought to regulate food intake and body weight [40]. Disruption of this receptor by a site-specific gene knock-out led to late onset obesity, hyperphagia, hyperinsulinemia and hyperglycemia. Sequence analysis also demonstrated a relatively high frequency of pathogenic mutations in the MC4R gene in individuals with severe early-onset obesity, which indicates that MC4R mutations are one of the most common monogenic causes of obesity in humans [49]. MC4R knock-out mice fed the HFC diet for nearly 5 months developed significant steatohepatitis, fibrosis, insulin resistance and hyperlipidemia, and the severity of these parameters was much worse compared to wildtype mice with the same diet. When the feeding was extended to 12 months, in addition to the more extensive fibrosis, all mice developed multiple and well-differentiated tumors in their livers, and tumor tissue was α-FP positive, and it exhibited severe dysplasia and fat-droplet accumulation [40]. It was recently reported that eicosapentaenoic acid (EPA) could prevent HFC diet-induced NASH in this model by reducing macrophage-associated inflammation, steatohepatitis (apoptosis) and fibrosis after the development of NASH [50]. This is another example of antioxidants (EPA and silymarin) in improving NASH in a rodent model [50, 51]; however, whether it is beneficial in preventing HCC development requires a long-term experiment.

#### Mice with metabolic iron deficiency

129S1/SvImJ mice are deficient in iron metabolism and were used for the development of NASH. It was demonstrated that feeding mice choline/folate-deficient diet resulted in their developing histopathologic features of NASH, and there exists a close correlation between the extent of liver injury and the abnormal expression of iron metabolic genes, such as transferrin receptor, ferritin heavy chain, solute carrier family 40 (iron-regulated transporter), member 1 (Slc40a1, Fpn1) and their related proteins, and pronounced down-regulation of the iron regulatory protein 1 (IRP1). These presentations indicate that abnormal iron metabolism-associated oxidant stress affects the rate and severity of hepatic necroinflammatory responses in NASH development [41]. In a recent study the same strain of mice was used for induction of liver malignancies by HFC diet plus high fructose/glucose (HFG) for 52 weeks. The mice fed the HFC diet plus HFG displayed the highest NASH activity and fibrotic scores, and at the same time, 8 out of 9 mice in this group developed HCC or hepatic adenoma, which was higher than in (6/15) those fed the HFC diet alone (2014 AASLD annual meeting Abstract #1097. Hepatology 2014; 60: 734A). Thus, it implies that the combination of high fat diet with high fructose/glucose in drinking water will be more effective in induction of insulin resistance, lipotoxic inflammatory and fibrotic responses, and drive malignant transformation in this strain of mice with iron metabolic deficiency. Although the focus of these studies does not emphasize the critical role of iron overload in development of steatosis and HCC, it is well documented that iron overload is a pivotal factor leading to hepatocellular damage and malignant transformation [52].

### Rodent models of NASH-HCC caused by a variety of diet feeding

#### Mice fed MCD diet

Methionine/choline deficient (MCD) diet has been long used for chronic liver injury, steatohepatitis, fibrosis and tumorigenesis due to the fact that methionine is an essential amino acid, and rodents are unable to synthesize it *de novo* [53]. Both methionine and choline are the precursor of phosphatidylcholine which is the critical component of VLDL. Lack of methionine and choline causes hepatocytes to be unable to secrete triglycerides in the form of VLDL, and triglycerides accumulate in the hepatocytes [35]. Methionine is an important methyl group donor and the lack of methionine gives rise to aberrant DNA and protein synthesis, and potentiates malignant transformation. The lack of methionine also hampers glutathione synthesis, and leads to S-adenosyl-L-methionine (SAMe) depletion and oxidant stress [54]. In addition, an excess of sucrose and trans-fat is supplemented in the formula of the MCD diet. These components exacerbated fat accumulation and hepatocellular damage of MCD diet-fed mice within 2-4 weeks [35, 55, 56]. Prolonged MCD feeding resulted in fibrosis (8 weeks) and the appearance of malignant neoplastic nodules in 3-6 months [55]. The tumorigenicity may be potentiated by exposure to DEN during MCD diet feeding [55]. Notably, the body weight of MCD diet-fed mice is often lower than controls, and there is no insulin resistance. Therefore, this model does not truly reflect metabolic syndrome-associated steatohepatitis but represents nutrient deficiency-associated steatohepatitis and subsequent fibrotic response and malignant transformation [35, 37]. Feeding mice the MCD diet not only increased total hepatic fatty acids and cholesterol, but also markedly elevated the C18/C16 ratio; the elongation of fatty acids from C16 to C18 has been shown to promote both hepatic lipid accumulation and inflammation. This finding is in accordance with DEN-induced carcinogenesis, in which increased hepatic lipids and the C18/C16 ratio were documented 48 hours after DEN exposure [57].

#### STAM model of NASH-fibrosis-HCC

A Japanese group developed a STAM mouse model [58] in which sequential development of steatohepatitis, fibrosis and carcinoma was achieved by starting treatment with streptozotocin at day 2 and feeding the HFC diet at 4 weeks after birth, and then NASH occurs at 8 weeks and cancer at 16-20 weeks [58, 59]. The advantage of this model is that it mimics the natural progression from steatohepatitis and fibrosis to HCC formation in diabetic mice during a relatively short period in a controllable fashion. The disadvantage is the lack of insulin resistance, and that these mice are not obese, but display significant diabetic alterations because of pancreatic islet damage by streptozotocin treatment [58]. It is also notable that female mice undergoing the same treatment did not develop NASH, fibrosis and HCC, which indicates that estrogen may play a protective role in susceptibility to NASH initiation and progression, as well as its malignant transformation in diabetic mice [58]. This group further characterized the tumor nodule size and metastatic status at 20 weeks of induction, and claimed that the stage of HCC seen in these mice corresponds to stages B to C of the Barcelona Clinic Liver Cancer (BCLC) staging system for humans [60]. This stratification could be helpful in assessing the efficacy of a therapeutic algorithm in the treatment of liver cancer with this model. This model has been used to test the effects of a liver-protective agent, silymarin (milk thistle extract), on steatosis [51].

#### Rat model of steatosis, fibrosis/cirrhosis and HCC

Rats were fed a choline-deficient plus high trans-fat diet for up to 16 weeks. Concomitantly, DEN was added into drinking water at 135 mg/L. At the end of the experiment, all 7 rats developed steatosis, fibrosis (7/7) and cirrhosis (6/7). HCC nodules were seen in all rat livers, and one was diagnosed as hepato-cholangio carcinoma. This is a typical NASH-fibrosis/cirrhosis-HCC model induced by trans-fat diet plus the carcinogen (DEN) [42]. The advantage of this model is that HCC is developed in a relatively short period compared to those without DEN intoxication. DEN is a known carcinogen that causes significant oxidant stress and DNA mutation, and it potentiates lipotoxicity and accelerates progression of fibrosis and cirrhosis [61]. Thus, this model does not reflect the natural malignant transformation process from NASH to HCC although there was striking activation of liver progenitor cells in the mixture of lipotoxicity and carcinogen exposure.

Approximately 55 % of rats fed the choline-deficient amino acid-defined (CDAD) diet for 48 weeks developed HCC on the background of NASH progression to cirrhosis. When a concurrent administration of an angiotensin II type 1 receptor blocker (ARB), telmisartan (2 mg/kg/day) during the last 24 weeks of CDAD feeding, fibrosis progression in these rats was strikingly minimized as demonstrated in histology, biochemical and molecular analysis. At the same time, no HCC occurred in this group [62]. The reduction of the HCC development rate in telmisartan-treated rats was attributed to the suppressive role of ARB on fibrosis as proven in other studies [63, 64], and suggests a possible strategy in the prevention of HCC development in NASH; there are no clinical studies yet using this approach.

#### American (sedentary) lifestyle-induced obesity syndrome (ALIOS) model

It is commonly accepted that NAFLD is closely associated with obesity. However, not all NAFLD patients are obese, and notably severe NASH may develop in patients with a normal basic metabolic index (BMI), indicating that the factors causing NAFLD pathogenesis are not well defined. Given the fact that a custom diet containing trans-fatty acids and high-fructose corn syrup (HFCS) plus sedentary lifestyle for 16 weeks produced a typical feature of NASH with obvious inflammation and fibrosis in mice [65], an extension of this approach to 12 months created an American (sedentary) lifestyle-induced obesity syndrome (ALIOS) model that accurately represents a broad similarity to the pathogenesis of human NASH and carcinoma [38]. The sedentary lifestyle was mimicked by providing drinking water as gel-water (93% water, 2.8% gelatin and 4.2% HFCS) in dishes on the cage floor. This allowed removal of cage racks in order to discourage physical activity in the ALIOS mice [65]. By the end of 12-month of feeding and a sedentary lifestyle, the comparison of NASH manifestations between humans and mice demonstrated an extensive similarity in insulin resistance, histopathology, adipokine and cytokine profile, as well as semi-quantitative scores of hepatic inflammation, steatosis and fibrosis. Moreover, 50% of mice developed perivascular hepatocellular neoplasms (half is microscopic) which are Sox-9-, β-catinen- or α-FP-positive [38]. Based on staining of these markers, expansion of hepatic progenitor cells was identified in the portal tract. In summary, this model truly represents a natural course of NASH-fibrosis/cirrhosis-HCC progression. The combination of sedentary lifestyle, striking necroinflammatory evidence, obvious insulin resistance, clear histopathologic features and appearance of macroscopic or microscopic hepatocellular neoplasms from a regular mouse strain (C57BL/6) are superior to other models in which carcinogens (DEN) were supplemented. The drawback of the study is the inclusion of only male mice since there is a marked difference in genders prone to lipotoxicity and steatotic progression [58].

#### Multiple gene disruption to prove T cell-mediated immune action in the development of NASH and its transition to HCC

It has been questioned whether lymphocytes, especially T lymphocyte-mediated adaptive immunity is involved in NASH development and its transition to HCC. In order to test this hypothesis, a group of scientists from Switzerland and Germany first confirmed that a 12-month choline-deficient and high fat diet (CD-HFD) caused NASH and HCC in 19 of 75 mice, which was much higher than HFD only in C57BL/6 mice [43]. After that, they employed several lines of knock-out mice to dissect the crucial role of CD8+ and NKT cells in NASH and HCC development, and found that CD-HFD activated intrahepatic CD8+ T and NKT cells, and that activated T lymphocytes released inflammatory cytokines and lymphotoxins, such as NKT-derived LIGHT (TNFSF, e.g., Light and Ltαβ) acting on hepatocytes, and exacerbating liver damage, NASH and HCC development. The lines of mice with genetic manipulation included Rag1 (lacking B, T and NKT cells) and β2m knock-out mice that lacked CD8+ and NKT cells. In addition, a line of CCR2 knock-out mice lacked proinflammatory monocytes and myeloid-derived B cells showed a similar frequency of HCC compared to wildtype mice. Moreover, hepatocyte-specific knock-out of canonical ikkβ^Δhep^ and LTβR signaling (LtβR^Δhep^ mice) confirmed that LTβR and NF-κB signaling in hepatocytes facilitated CD-HFD-induced liver cancer [43]. The study further verified that enhanced LIGHT-expressing CD8+ and NKT cells were identified in NASH and NASH-HCC specimens, which is in accordance with the findings from knock-out mice [43]. This is the first direct evidence demonstrating that adaptive immunity from CD8+ T cells and NKT cells is critical for the development of NASH and its transition to HCC through lymphotoxins, such as LIGHT signaling and NF-κB signaling in ballooned hepatocytes [43]. The findings also suggest that interference with localization of lymphocytes to the liver or blocking hepatocyte-lymphocyte cross-talk could be a promising strategy to treat NASH and prevent NASH-driven HCC [43]. This strategy is supported by a different study in animals deficient in glycine N-methyltransferase (GNMT), which catabolizes SAMe, the main methyl donor of the body [64].

#### ER stress in NASH progression and HCC development

In fatty acid-mediated lipotoxicity ER stress plays a crucial role *via* oxidant stress in the mediation of cell death through apoptosis and insulin resistance [66]. The perturbation of unfolded protein accumulation in ER stress causes ballooning degeneration of hepatocytes, a classical sign of NASH; thus, it is obvious that ER stress is a critical pathologic component for injury of steatotic hepatocytes in NASH initiation and progression [66, 67]. However, it is unclear whether ER stress also participates in hepatic carcinogenesis in NASH. In order to answer this question, a line of hepatocyte-specific MUP-uPA knock-out mice in which the MUP silencing was under the control of urokinase plasminogen activator (uPA) was generated and fed high fat diet for 40 weeks. Nearly 80% MUP null mice developed either hepatic adenoma or HCC in multiple nodular lesions [68]. MUP is a key molecule in maintaining protein folding and exclusion process in ER, and its site-specific deletion results in ER stress in hepatocytes. Much fewer (30%) adenomas or HCC nodules were seen in MUP-uPA KO mice with low fat diet feeding, and no tumor was found in control mice with low or high fat diet [68]. Other findings of the study further confirmed that MUP deletion enhanced lipogenesis, accelerated NASH progression, and promoted the oncogenic process. Further deletion of TNF-receptor 1 (TNF-R1) in MUP-uPA mice attenuated NASH progression and reduced HCC development in the same strain of mice, in which p65 signaling activity was reduced too. Therefore, it is proposed that TNF-α and NF-κB signaling is involved in ER stress-mediated lipotoxicity and hepatocellular death, as well as further transformation to malignancy [68]. To further support the critical role of ER stress in NASH progression and HCC development, knock-outs of another key protein in a gatekeeper, Gp78, an E3 ubiquitin ligase, which degrades unfolded protein in ER, resulted in up-regulation of unfolded protein response (UPR) pathways and SREBP-1 regulating *de novo* lipogenesis; the model spontaneously developed NASH, and further progressed to HCC in one year [69].

### The use of implanted xenografts to investigate the promotion of tumor proliferation and metastases by fatty liver

Not only does fatty liver provide a microenvironment for oncogenesis of hepatic or cholangio-carcinoma, but also it is rich soil fostering tumor growth and metastasis [70]. It has been speculated that injury of hepatocytes and subsequent inflammatory cytokines, adipokines, and growth factors released by parenchymal or non-parenchymal cells promote tumor growth and metastasis. To prove this speculation, hepatoma cells were injected into fatty liver caused by choline-deficient diet for 6 weeks through the portal vein in rats, and metastasis of these hepatoma cells was markedly enhanced in fatty liver compared to those injected into normal livers. Co-implanted hepatoma cells with hepatic stellate cells (HSC) isolated from fatty liver promoted the growth of xenografts compared to those co-implanted with HSC from normal livers, which is attributed to the release of cytokines such as vascular endothelial growth factor (VEGF), interleukin-1α (IL-1α) and transforming growth factor-β (TGF-β) from *in situ* activated HSCs in fatty liver [71]. However, conflicting results were acquired in a different study [72], which claims that feeding rats with saturated fat diet for 10-12 weeks delayed the initiation of DEN-induced hepatic carcinogenesis. Apparently, these two studies had different focuses and experimental designs; therefore, more studies are needed to address emerging questions in better defined conditions.

## PROSPECTIVE AND CONCLUSIONS

It has been estimated that more than 2 billion individuals worldwide are affected by obesity, diabetes, metabolic syndrome and other lipid metabolic disorders. The speculation is that 10-36% of the general population suffers from various stages of NAFLD [1, 2]. When one of 5-10 NAFLD patients progress to NASH, subsequently to ESLD and HCC, it is not surprising that NASH-associated ESLD and HCC have already become the 2^nd^ leading etiology for liver transplantation in the US [10, 73]. Therefore, NASH-associated ESLD and HCC will gradually become the major etiology of liver-related mortality worldwide when many HCV patients are cured with direct-acting antiviral (DAA) agents [74], HBV vaccination is successful, and infected HBV patients are treated effectively [75].

### Dilemma of NASH-HCC

Currently, hepatic carcinogenesis is not fully understood in general, and HCC management strategies are not effective enough to significantly increase 5-year survival rate [69]. Clinical and laboratory research of NASH-associated HCC is in its infancy, and faces tremendous challenges, such as multifactorial features, difficulty in establishing causative-effect links in population studies, and lack of reliable animal models for NASH and NASH-HCC. This phenomenon is especially common in regions with a higher HBV or HCV infection rate, where it is believed that the incident of HCC derived from NAFLD/NASH is very low because when an individual is HBV or HCV-positive, HCC is attributed to be the consequence of viral infection whether NAFLD/NASH is present or not [76].

Animal models are inadequate to completely unveil how HCC occurs in NASH at the molecular, genetic or epigenetic levels, nor does there exist an adequate NASH-HCC rodent model. From the findings of currently available NASH-HCC models, it gradually becomes clear that factors that affect NASH progression largely contribute to HCC development, and which certainly correlates with the degree of liver damage as a result of lipotoxicity through oxidant stress [63, 77, 78], ER stress [44], autophagy [28] or adaptive immunity [43]. Hepatocellular injury elicits a regenerative response of hepatocytes and recruitment of progenitor cells, both of which may be abnormal due to disturbed profiles of cytokines, adipokines and lymphokines in a steatotic and fibrotic micro-environment [30]. Hepatic fibrogenesis may facilitate the development and metastases of HCC by providing growth factors and interstitial niche of oncogenesis [22, 79]. Gut microbiota may also contribute to steatotic and oncogenic processes through abnormal bile acid metabolism and intrahepatic inflammation and senescence of HSCs [19]. Therefore, NASH is rich soil for HCC development, and when it progresses to cirrhosis, the rate of HCC occurrence is accelerated in an even worse microenvironment that fosters aberrant proliferation (dysplasia) and malignant transformation [29]. Such a complicated and dynamic phenomenon can only be mimicked by animal models although they do not exactly resemble the patterns, dietary profiles, milestones of NASH → Fibrosis → HCC progression, as well as prognosis and complications of the disease in humans in a single model. It is clear, however, that these animal models are valuable tools to recapitulate the complicated processes and to dissect the roles of critical genes and pathways of multiple factors, and that they are supplemental to clinical investigations [19, 43, 68]. *In vitro* approaches will be insufficient to evaluate potential preventive and therapeutic strategies to reverse NASH and block the transition to HCC. Such an intervention might include supplementation with choline [33], eicosapentaenoic acid (EPA) [29, 50], silymarin [51], SAMe [62], coffee [80], coumarin [81] or even a hedgehog signaling inhibitor vismodegib [82], and PPAR-α, δ or γ agonists [83] are all therapeutic possibilities based on preclinical studies. Vitamin E [84], FXR agonists (obeticholic acid) [85] and insulin-sensitizing medications (pioglitazone) [86, 87] are in clinical use. An extensive review of pharmacologic action, efficacy and adverse effects of these potential therapeutics are beyond the theme of this review. To date the clinical trials for NAFLD have not been as successful as recent trials for viral hepatitis probably due to the fact that there are no etiology-specific therapeutics available for NASH. Multiple-center randomized trial data are available in demonstrating that the effective anti-HBV nucleotides or anti-HCV DAAs not only reverse the extent of fibrosis, even early cirrhosis, but also decrease HCC incidence in HBV or HCV-infected individuals [75, 79, 88, 89].

### Selection of NASH-HCC animal models

Currently available rodent NASH-HCC models are basically an attempt to investigate NASH progression with or without a carcinogen exposure (Table 1). Genetic manipulation is a reliable tool to define the role of a single gene or combination of genes in pathophysiologic function, molecular basis of disease progression and therapeutic strategies, such as a tumor suppressor gene (PTEN) [45], and a promoting factor of liver regeneration, argumenter of liver regeneration (ALR) [48]. It was paradigm-shifting to use a series of knock-out mouse lines to specifically delete subsets of T cells, such as CD8+ T and NKT cells, in order to prove that adaptive immunity plays a critical role in the development of NASH and its transition to HCC [43]. The verification of ER stress contribution to NASH progression and HCC development in MUP-uPA knock-out mice highlights the importance of lipotoxicity and hepatocellular death as promoting factors in these processes [68]. The use of a specific mouse strain with iron metabolic deficiency may shorten the duration of NASH development and progression to HCC. The STAM model has a sequential progression from NASH → fibrosis → HCC during a relatively short duration and in a controllable fashion in diabetic mice without significant insulin resistance [58]. The MCD diet induces steatohepatitis, fibrosis and HCC in non-obese mice with no significant insulin resistance [55]. CD-HFD predisposes mice to an increased frequency of HCC development compared to high fat diet alone in one-year term of feeding [43]. Sedentary ALIOS model resembles Westernized lifestyle, clinical features and pathophysiologic progression of NASH→fibrosis→HCC to a great extent, and could be considered as a useful natural course of HCC development in NASH progression [38]. Concomitant addition of a carcinogen, such as DEN to HFC or trans-fat plus high fructose diet potentiates the oncogenic toxicity, and may shorten the induction duration, but adds extra layers of complexity in carcinogen-induced genomic instability and metabolic impacts [42, 55]. Thus, selection of a suitable animal model of NASH-HCC depends on specific questions to be addressed, and resources and technologies available to seek answers. Moreover, the findings from one type of animal models often require additional verification in a different model, and there is significant difference in susceptibility to NASH and HCC development between genders.

**Table 1:** Rodent models of NASH-associated HCC

Name of models	Species or genetic background	Diet or agents used to induce NAFLD/NASH	Inducing duration	Description of hepatic neoplasms
PTEN-null mice [45–47]	AlbCrePten^flox/flox^ mice	Spontaneous formation of NASH and HCC	NASH in 10 wks and HCC in 40 wks.	Hepatocellular and/or cholangio-carcinoma
ALR-null mice [48]	AlbCre ALR^flox/flox^ mice	Spontaneous formation of liver inflammation, steatosis and neoplasms	Necroinflammation at 4 wks, steatosis, fibrosis at 8 wks and HCC at 1 year.	Nodular foci of high-grade dysplasia after 6 months, liver tumors in 70% of mice within 1 year, and 60% of them are HCC.
MC4R-deficient mice [40]	MC4R-knock-out strain	HFC diet for 12 months	5 months for NASH. 12 months for HCC	Well-differentiated HCC seen in 5/5 mice at 12 months of feeding.
Mice fed CD-HFD [43]	Regular mouse strain	Choline-deficient high fat diet	NASH develops at 5-6 months, HCC develops at 12 months	HCC in 19 of 75 mice within 1 year of induction. Tumor types are heterogeneous.
Mice with iron metabolism defect [41]	129S1/SvImJ strain	HFC diet plus high fructose and glucose in drinking water	52 wks	HCC in 8/9 mice, rest with hepatic adenoma.
Mice fed MCD diet [35, 55]	Regular mouse strains	Methionine/choline deficient diet	4-6 months	Steatohepatitis at 4 wks, fibrosis at 8 wks, and HCC at 20-26 wks.
STAM model of NASH-fibrosis-HCC [58, 59]	New born mice	Sequential induction with streptozotocin and HFC diet	NASH occurs at 8 wks, and HCC at 20 wks	HCC in diabetic mice without insulin resistance
Rat model of steatosis, fibrosis/cirrhosis and HCC [42]	Rats	Choline-deficient plus high trans-fat diet, DEN in drinking water	16 wks	Steatohepatitis & fibrosis in 7/7, cirrhosis in 6/7, HCC in 7/7, and cholangio-carcinoma in 1/7
Rat model of NASH-cirrhosis-HCC [62]	Rats	Choline-deficient amino acid-defined diet	48 weeks	54.6% rats developed HCC on the basis of cirrhosis during 48 weeks of feeding. HCC is positive for HIF-α1 and VEGF.
Sedentary ALIOS model [38, 65]	Mouse	A custom diet containing trans-fatty acids and HFCS plus sedentary lifestyle	Typical NASH at 16 wks, 12 months for an advanced stage	NASH, fibrosis/cirrhosis in all mice. 50% developed neoplasms with half microscopic lesions with evidence of progenitor activation.

ALIOS = American (sedentary) lifestyle-induced obesity syndrome; ALR = argumenter of liver regeneration; CD-HFD = choline-deficient high fat diet; DEN = diethylnitrosamine; HFCS = high-fructose corn syrup; HFC = high fat/high Clarrie; HIF-1α = hypoxia-inducible factor-1α; MCD = methionine/choline deficient; MC4R = Melanocortin 4 receptor; PTEN = Phosphatase and tensin homolog; VEGF = vascular endothelial growth factor; wks = weeks.

### Remaining issues that can be addressed by NASH-HCC animal models

There are a series of unsolved issues involved in the pathophysiology of NASH-HCC that can be addressed employing animal models. One unsolved question remains whether cancer stem cells or tumor-initiating cells arise from transformation of normal tissue stem cells, or from dedifferentiation of mature hepatocytes or progenitor cells during steatohepatitis [90]. Other intriguing unsolved issues include, but are not limited to: 1) The impact of altered gut microbiome on bile acid metabolism and hepatic inflammation, steatosis and carcinogenesis [17]; 2) What is the epigenetic influence from aberrant adipokines, lymphokines, cytokines or transcription factors on normal stem cells, progenitor cells or mature hepatocytes [30, 90]; 3) Are there differences in energy and nutrient metabolism and the metabolomics profile between SFL, NASH and NASH-HCC; and 4) The molecular links between disordered lipid metabolism, ER stress, and insulin resistance and carcinogenesis [91]. Hopefully, reliable NASH-HCC models will be available in the near future which will enable these studies to be undertaken, and also be used to assess the effectiveness of potential therapeutics in the treatment of NASH and the prevention of HCC [46, 51].

## Abbreviations

ALR: argumenter of liver regeneration
ALIOS: American (sedentary) lifestyle-induced obesity syndrome
DAA: direct-acting antiviral
DEN: diethylnitrosamine
EPA: eicosapentaenoic acid
ESLD: end-stage liver disease
HCC: hepatocellular carcinoma
HFC: high fat/high Calorie
HFCS: high fructose corn syrup
HSCs: hepatic stellate cells
MCD: methionine/choline-deficient
NAFLD: nonalcoholic fatty liver disease
NASH: nonalcoholic steatohepatitis
NASH-HCC: NASH-associated hepatocellular carcinoma
PTEN: phosphatase and tensin homolog
PUFA: poly non-saturated fatty acids
ROS: reactive oxygen species
SAMe: S-adenosylmethionine
SFL: simple fatty liver.
